# Association between dietary trace minerals and pelvic inflammatory disease: data from the 2015–2018 National Health and Nutrition Examination Surveys

**DOI:** 10.3389/fnut.2023.1273509

**Published:** 2023-11-27

**Authors:** Panwei Hu, Siming Zhang, Haoyuan Li, Xiaotong Yan, Xiaole Zhang, Qinhua Zhang

**Affiliations:** ^1^Department of Obstetrics and Gynecology, Shuguang Hospital Affiliated to Shanghai University of Traditional Chinese Medicine, Shanghai, China; ^2^Department of Obstetrics and Gynecology, Shanghai Fengxian District Traditional Chinese Medicine Hospital, Shanghai, China

**Keywords:** pelvic inflammatory disease, dietary trace minerals, dietary copper intake, National Health and Nutrition Examination Survey, cross-sectional study

## Abstract

**Objective:**

Pelvic inflammatory disease (PID) is a prevalent gynecological disorder. Dietary trace minerals play an important role in combating many chronic diseases including PID. However, it is unknown whether dietary trace minerals and PID are related. This study aimed to examine the relationship between dietary trace minerals (copper, iron, selenium, and zinc) and PID.

**Methods:**

Data of women participants from the National Health and Nutrition Examination Survey (NHANES) 2015–2018 were enrolled in this cross-sectional investigation. Univariate and multivariate linear regression analyses of the relationship between dietary trace minerals and PID were performed, and restricted cubic spline (RCS) analyses were applied to visualize those relationships.

**Results:**

In total, 2,694 women between the ages of 20 and 59 years participated in the two NHANES cycles. In the univariate analyses, a significant negative relationship was identified between PID and dietary copper intake [odds ratio (OR) = 0.40, 95% confidence interval (CI): 0.24–0.67, *p* < 0.01] but not with iron (OR = 0.96, 95% CI: 0.90–1.03, *p* = 0.25), selenium (OR = 1.0, 95% CI: 0.99–1.0, *p* = 0.23), and zinc (OR = 0.94, 95% CI: 0.86–1.03, *p* = 0.17) intake. Following the adjustment for age and race (model 1), a robust correlation was found between dietary copper intake and PID (OR = 0.23, 95% CI = 0.09–0.61, *p* < 0.01), as indicated by the fully adjusted model 2 (OR = 0.29, 95% CI = 0.09–0.90, *p* = 0.03). Simultaneously, a significant trend was found between copper intake and PID across the quintile subgroups (*p* for trends <0.05), suggesting a robust relationship. Furthermore, the RCS analysis demonstrated a linear correlation between PID and dietary copper intake (overall *p* < 0.01, non-linear *p* = 0.09).

**Conclusion:**

Decreased dietary copper intakes are linked to PID. However, additional research is needed to fully investigate this relationship due to the constraints of the study design.

## 1 Introduction

Pelvic inflammatory disease (PID) is a polymicrobial infection that generally occurs in the women’s genital tract and often leads to the impairment of the endometrium, oviduct, and ovaries (1). The clinical manifestations of PID include long-term infertility, extrauterine pregnancies, and chronic pelvic pain (2). The cause of PID is associated with many factors, including race, age, and smoking status (3). Young people and African or Caucasian people are especially prone to PID (4). A study has estimated that 4 to 12% of women of reproductive age have PID worldwide (5). However, few studies on the epidemiological trends of PID among different areas of the world have been conducted due to challenges with the invasiveness and sensitivity of screening methods (6). In the United States, approximately 0.5 to 1 million cases of PID develop annually, and the average cost is up to $3,025 per episode for PID therapy (7, 8). At the same time, the majority of PID patients are prone to experience disease recurrence, resulting in additional burdens on society and healthcare systems (9). Hence, it is crucial to examine the risk factors associated with PID to enable early intervention.

Diets enriched with antioxidants (fruit and vegetables), vitamins (vitamins B6, A, C, E), and macronutrients (n-3 fatty acids) improve immune system functioning and eliminate free radical damage, which can help control chronic pelvic pain, including PID (10). Although trace minerals account for only a small part of the diet, they are essential to ensure normal function and health, and the excessive or inadequate consumption of trace elements is detrimental to the inherent metabolic balance of the body (11). Trace elements, including copper, iron, selenium, and zinc, are vital for maintaining metabolic balance, especially in cell function, DNA repair, and antioxidant defense (12). Previous studies have indicated that dietary trace minerals have powerful regulatory effects on the oxidative activation of protein kinase C, prostaglandin synthesis, and Ca^2+^ receptors which, in turn, increase chronic pain intensity (13). A study has revealed that copper is closely related to inflammatory responses in many chronic diseases, such as metabolic disorders and cancer (14). Additional studies have illustrated that the intake of dietary copper and selenium is linked to many diseases including gynecological tumors and metabolic syndrome (15–17). Meanwhile, dietary intake of iron and zinc was found to be negatively correlated with fibromyalgia, characterized by widespread muscle soreness, and other chronic symptoms such as fatigue, anxiety, and depression (18). However, the relationship between dietary trace elements and PID is not fully understood.

To this end, using data from the NHANES, the current study aimed to determine if dietary trace elements and PID were correlated after controlling for covariates.

## 2 Methods

### 2.1 Study design and participants

**Figure 1:**
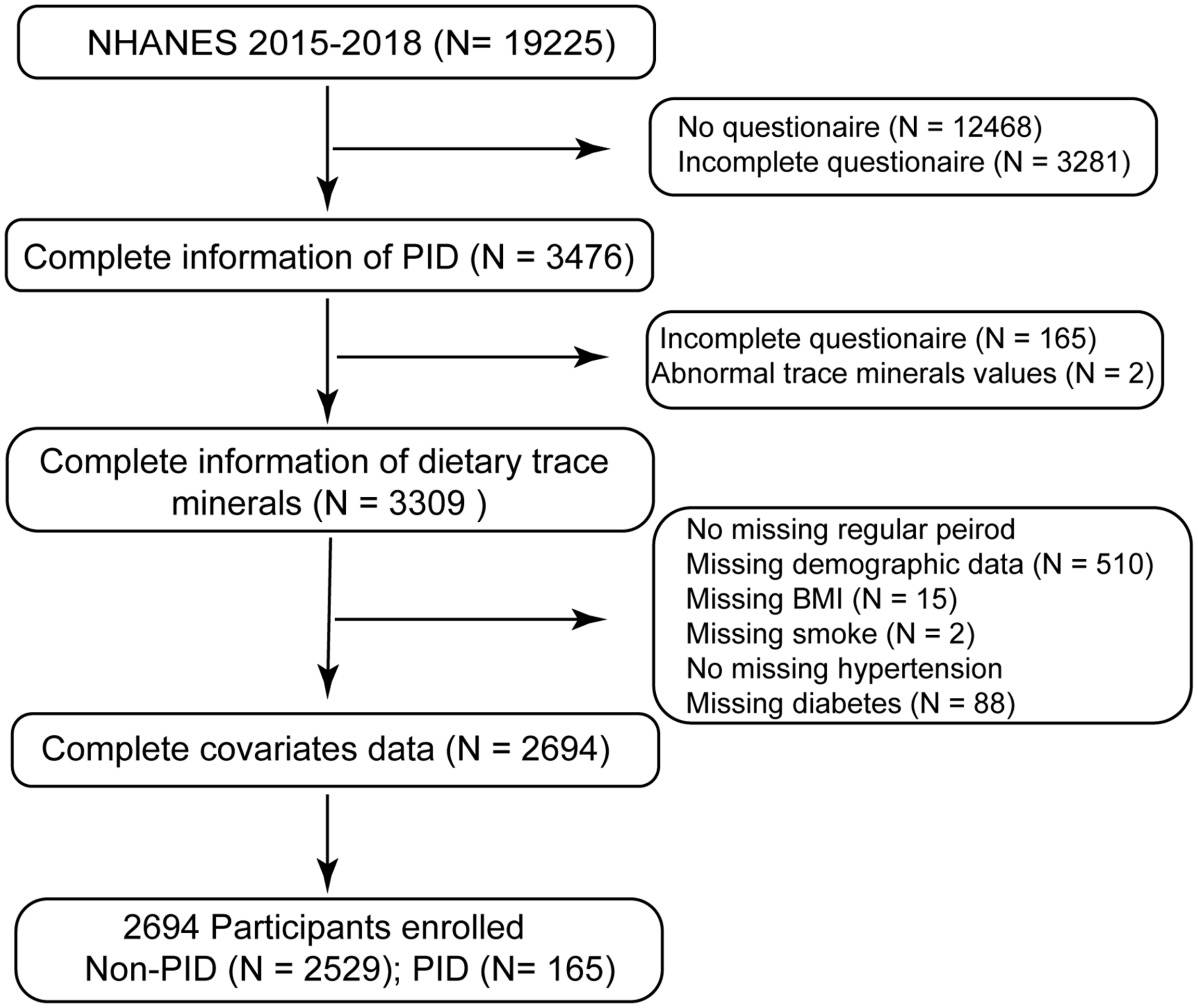
Screening procedure flowchart (NHANES 2015–2018).

Based on the large sample size of the NHANES, data from 2015 to 2018 (two cycles: 2015–2016 and 2017–2018) before the coronavirus disease in 2019 were selected for analysis. Since PID occurs in women’s genital tracts, only females were recruited. All women participants were initially interviewed at home using the sample person and family demographics questionnaires, followed by either another interview or health examination at a mobile examination center (MEC). During these MEC examinations, participants were screened for direct anthropometrics, height, weight, blood draws, and various other health-related tests (19). Before the study, all subjects provided written informed consent, and no external ethical approval was needed since the research was approved by the National Center for Health Statistics Ethics Review Board (https://www.cdc.gov/nchs/nhanes/irba98.htm, continuation of protocol #2011–17, protocol #2018–01). The analysis only included women participants whose data were complete. We excluded 15,749 participants with missing PID, 167 with missing dietary trace minerals, and 615 missing covariates from the 19,225 eligible individuals. This research eventually included 2,694 individuals aged 20–59 years from the entire US population. The original NHANES study was a random sampling trial, and our research adopted a cross-sectional method based on the NHANES database. The women’s selection scheme is shown in Figure 1. At the same time, our study was weighted to allow the representation of the entire population in the US.

### 2.2 Measurement of PID

Based on a self-reported questionnaire of reproductive health, the diagnosis of PID was ascertained at the MEC by trained interviewers who used the built-in Computer-Assisted Personal Interview (CAPI) system, which enquired, ‘Have you ever been treated for an infection in the fallopian tubes, uterus, or ovaries, also called a pelvic infection, pelvic inflammatory disease, or PID?’. A “PID group” was formed from participants who replied “yes” whereas a “without PID group” was formed from those who answered “no” (20).

### 2.3 Measurement of dietary trace minerals

The dietary intake data was acquired at the MEC through standard dietary interviews, called What We Eat in America (WWEIA), and types and amounts of food and beverages consumed during the 24-h period immediately prior to the interview were obtained. WWEIA interviews were randomly allocated to the participants on the day of the interview. Before they could start working independently in the MEC, all dietary interviewers were asked to complete a standardized training course and engage in practice interviews under supervision. Finally, the daily total intakes of trace minerals (copper, iron, selenium, and zinc) from foods and beverages were then collated to obtain daily trace intake data. By using the recommended dietary allowance (RDA) in women (0.9 mg/day for copper, 18 mg/day for iron, 55 mcg/day for selenium, and 11 mg/day for zinc) (21), values of dietary trace minerals over 10-fold of the RDA were excluded as outliers.

### 2.4 Assessment of covariates

Based on related studies (20, 22), regular period, democratic data (race, education level, age, marital status, and poverty), BMI, smoking status, diabetes mellitus (DM), and hypertension were treated as covariates. Regular period data were assessed through the reproductive health questionnaire that asked, ‘Had regular periods in past 12 months (eligible for participants aged 12 years older)?’, and women who were pregnant or who had bleeding as result of medical issues, hormonal treatments, or surgical procedures were excluded. The CAPI method was used to collect information from in-depth interviews with participants in their homes conducted by trained interviewers. Participants in the survey answered these questions using hand cards depicting response options or information. The interviewer directed the respondent to the appropriate hand card during the interview, and the complete data of democratic covariates was obtained. Educational levels were classified as lower than high school, high school, and college, whereas marital status was classified as married, spinsterhood, divorced/separated, or widowed. Subsequently, the poverty income ratio (PIR) was divided into three groups: low (≤ 1), medium (1–3), and high (> 3) (22). The data of weight and height were obtained electronically from the measuring devices to minimize potential data entry errors, and the individual’s body mass index (BMI) was obtained using the formula BMI = weight (kg)/height (m^2^). After that, BMI was categorized into: obese (≥ 30.0 kg/m^2^), overweight (25–29.9 kg/m^2^), normal weight (18.5–24.9 kg/m^2^), and underweight (< 18.5 kg/m^2^) (23). The CAPI system was used to conduct in-home interviews by trained interviewers who administered a “smoking-cigarette usage” questionnaire to determine whether respondents smoked and then assigned to three classes of smoking status: never (no more than 100 cigarettes in whole life), former (over 100 cigarettes in whole life and quit smoking right now), and now (over 100 cigarettes in whole life and smoke some days or every day). After resting for at least 5 minutes, the blood pressure of participants was measured three consecutive times. Repeated attempts were made to measure blood pressure if the first one was incomplete or interrupted. All systolic and diastolic blood pressure measurements were performed in the MEC, and hypertension was diagnosed based on the threshold of systolic/diastolic blood pressure at 140/90 mmHg. Blood specimens were processed, stored, and shipped to a specific site for testing the glucose. Women’s DM condition was classified as no DM, pre-DM (impaired glucose tolerance (IGT), impaired fasting glucose (IFG), or combined IFG and IGT), and DM (24).

### 2.5 Statistical analysis

R (v4.2.1) and R Studio (v2022.07.1) were employed to analyze statistical data. Dietary weights were adopted to ensure the rigor and accuracy of the study under the least common denominator strategy of sampling weight guidance of the NHANES. Categorical variables are presented as proportions (n) and percentages (%), whereas continuous variables are presented as mean and standard error (SE). Categorical and continuous characteristics were analyzed using the chi-squared tests and *t*-tests, respectively. To verify the correlation between dietary trace minerals and PID, dietary trace minerals that were shown to be statistically significant in univariate logistic regression analysis were then subjected to multivariate logistic regression analyses. Adjustments for age and race were made to Model 1, whereas adjustments for all other covariates were made to Model 2 (regular period, demographics, BMI, smoking status, hypertension, and DM). Subsequently, restricted cubic splines (RCS) were adopted to assess the linear and non-linear associations, and these relationships were tested using quintile logistic regression analyses. Subgroup analyses and interaction tests were conducted to evaluate potential interactions, and RCS was administered to determine how dietary trace minerals correlated with PID after stratification of meaningful interaction terms. The criterion for statistical significance was established at a two-tailed *p*-value <0.05.

## 3 Results

### 3.1 Description of participants

According to the study’s eligibility criteria, 2,694 female participants were enrolled, and the average age of the participants was 40 ± 0.4 years. Among these participants, 32.67% were non-Hispanic white, 23.01% were non-Hispanic black, 16.82% were Mexican American, and 27.51% were from other races. There were 2,529 (93.88%) participants without PID and 165 (6.12%) with PID. A detailed description of the women participants is provided in Table 1. Of those individuals with PID, the weighted mean concentration of dietary trace minerals was 0.95 mg/day (copper), 10.96 mg/day (iron), 89.9 mg/day (selenium), and 8.30 mg/day (zinc), respectively. There were 56 (54.79%) participants who were Non-Hispanic White, 96 (57.39%) had a college education, and 131 (86.8%) participants without diabetes. Participants with PID were more likely to have the following characteristics relative to those without the disorder: a lower copper intake (*p* < 0.01), irregular period (*p* < 0.01), older age (*p* < 0.01), non-married status (*p* < 0.01), better economic situation (*p* < 0.01), obesity or overweight (*p* < 0.01), history of no or current smoking status (*p* < 0.01), and hypertension (*p* < 0.01).

**Table 1:** Characteristics of the women participants [mean and standard errors (SE); proportions (n) and percentage (%)].

Characteristics	Total	Without PID (*n* = 2,529)	PID (*n* = 165)	*p*-value
Copper (mg/day)	1.12 (0.02)	1.14 (0.02)	0.91 (0.05)	< 0.01
Iron (mg/day)	12.31 (0.21)	12.40 (0.23)	10.96 (0.98)	0.18
Selenium (mcg/day)	99.18 (1.28)	99.77 (1.32)	89.90 (6.94)	0.17
Zinc (mg/day)	9.52 (0.16)	9.60 (0.16)	8.30 (0.76)	0.10
Regular period (*n*, %)				0.01
No	875 (32.48)	798 (35.23)	77 (48.93)	
Yes	1819 (67.52)	1731 (64.77)	88 (51.07)	
Age (years)	40.23 (0.40)	40.03 (0.42)	43.40 (1.13)	0.01
Race (*n*, %)				0.32
Non-Hispanic White	880 (32.67)	824 (60.60)	56 (54.79)	
Non-Hispanic Black	620 (23.01)	567 (11.74)	53 (16.63)	
Mexican American	453 (16.82)	435 (10.38)	18 (6.59)	
Other races	741 (27.51)	703 (17.28)	38 (21.99)	
Marital (*n*, %)				0.02
Married	1,567 (58.17)	1,479 (62.58)	88 (55.29)	
Widowed	59 (2.19)	53 (2.08)	6 (5.35)	
Divorced/separated	416 (15.44)	374 (13.28)	42 (25.80)	
Spinsterhood	652 (24.2)	623 (22.06)	29 (13.56)	
Education (*n*, %)				0.20
Less high school	398 (14.77)	370 (9.15)	28 (13.52)	
High school	560 (20.79)	519 (21.45)	41 (29.10)	
College	1736 (64.44)	1,640 (69.40)	96 (57.39)	
Poverty (*n*, %)				< 0.01
Low	597 (22.16)	546 (16.44)	51 (24.96)	
Medium	1,133 (42.06)	1,054 (33.92)	79 (51.69)	
High	964 (35.78)	929 (49.64)	35 (23.35)	
BMI (*n*, %)				< 0.01
Underweight	55 (2.04)	54 (2.52)	1 (0.17)	
Normal weight	745 (27.65)	712 (30.97)	33 (12.48)	
Overweight	660 (24.5)	618 (24.21)	42 (36.63)	
Obese	1,234 (45.81)	1,145 (42.31)	89 (50.73)	
Smoke (*n*, %)				< 0.01
Never	488 (18.11)	433 (18.19)	55 (37.30)	
Former	1825 (67.74)	1757 (66.36)	68 (36.09)	
Now	381 (14.14)	339 (15.45)	42 (26.61)	
Hypertension (*n*, %)				< 0.01
No	1942 (72.09)	1851 (77.10)	91 (62.44)	
Yes	752 (27.91)	678 (22.90)	74 (37.56)	
Diabetes mellitus (*n*, %)				0.55
No	2,186 (81.14)	2055 (83.39)	131 (86.80)	
Pre-diabetes mellitus	156 (5.79)	147 (5.45)	9 (4.57)	
Diabetes mellitus	352 (13.07)	327 (11.15)	25 (8.63)	

The format of mean ± standard error (SE) is used to present continuous variables, whereas counts and percentages are used to present categorical variables. Categorical and continuous characteristics were analyzed using the chi-squared tests and *t*-tests, respectively.

### 3.2 PID correlation with dietary trace minerals

The correlation between dietary copper intake and PID was substantially negative, as indicated in Table 2 (OR = 0.40, 95% CI: 0.24–0.67), whereas the same trend was not significant in iron (OR = 0.96, 95% CI: 0.90–1.03), selenium (OR = 1.0, 95% CI: 0.99–1.0), and zinc (OR = 0.94, 95% CI: 0.86–1.03). The correlation between dietary copper intake and PID was negative in a univariate analysis. Hence, multivariate logistic regression was used to delve deeper into the correlation between copper and PID. Dietary copper intake was shown to be significantly correlated with PID after adjusting for age and race (model 1) (OR = 0.41, 95% CI = 0.23–0.70). This association remained consistent in model 2 (OR = 0.51, 95% CI = 0.28–0.90) after adjusting for all covariates.

**Table 2:** Weighted univariate logistic analyses between dietary trace minerals and PID (odds ratios, 95% confidence intervals).

Dietary trace minerals	Univariate analysis (crude model)	
	95% CI	*p*
Copper (mg/day)	0.40 (0.24, 0.67)	< 0.01
Iron (mg/day)	0.96 (0.90, 1.03)	0.25
Selenium (mcg/day)	1.0 (0.99, 1.0)	0.23
Zinc (mg/day)	0.94 (0.86, 1.03)	0.17

### 3.3 Relationship between PID and various quintiles of dietary copper intake

**Figure 2:**
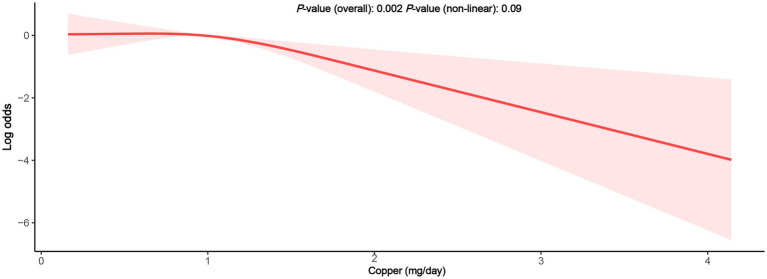
The RCS between dietary copper intake and the risk for PID.

When divided into quintiles (Table 3), the quintile of copper intake (Q5 > 1.49 mg/day) served a protective role in PID, regardless of the unadjusted or adjusted models (crude model: OR = 0.23, 95% CI = 0.09–0.58; model 1: OR = 0.23, 95% CI = 0.09–0.61; model 2: OR = 0.29, 95% CI = 0.09–0.90). Simultaneously, a significant trend was observed between copper intake and PID across the quintile subgroups (*p* for trend <0.05), suggesting the robustness of this relationship. To visualize the correlation between dietary copper intake and PID, an RCS plot was generated (Figure 2), demonstrating an approximately linear correlation between the two variables (overall *p*-value <0.01, non-linear *p*-value = 0.09).

**Table 3:** Weighted multivariate logistic analyses between quintiles of dietary copper intake and PID (odds ratios, 95% confidence intervals).

Dietary copper intake (mg/day)	Crude model		Model 1^a^		Model 2^b^	
	95% CI	*p*	95% CI	*p*	95% CI	*p*
Copper	0.40 (0.24, 0.67)	< 0.01	0.41 (0.23, 0.70)	< 0.01	0.51 (0.28, 0.90)	0.03
Q1 ≤ 0.63	Reference		Reference		Reference	
Q2 0.63–0.86	0.85 (0.40,1.81)	0.66	0.85 (0.39,1.85)	0.68	0.91 (0.39, 2.14)	0.80
Q3 0.87–1.09	0.67 (0.36,1.26)	0.20	0.69 (0.35,1.34)	0.26	0.83 (0.34, 2.03)	0.63
Q4 1.10–1.48	0.59 (0.28,1.27)	0.17	0.60 (0.27,1.34)	0.20	0.84 (0.32, 2.20)	0.68
Q5 > 1.49	0.23 (0.09,0.58)	< 0.01	0.23 (0.09,0.61)	0.01	0.29 (0.09, 0.90)	0.04
*P* for trend		< 0.01		< 0.01		0.04

There was no adjustment made to the crude model.

a Age and race adjustments were made to Model 1.

b All covariates (age, regular period, demography, BMI, smoke, hypertension, and DM) were adjusted to Model 2.

### 3.4 Subgroup analysis

**Figure 3:**
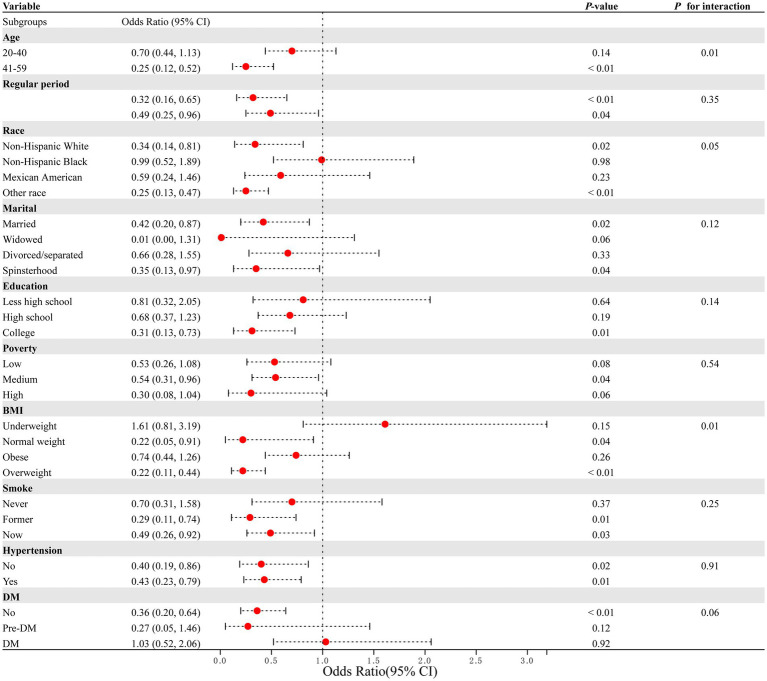
Subgroup analysis between dietary copper intake and the risk for PID.

**Figure 4:**
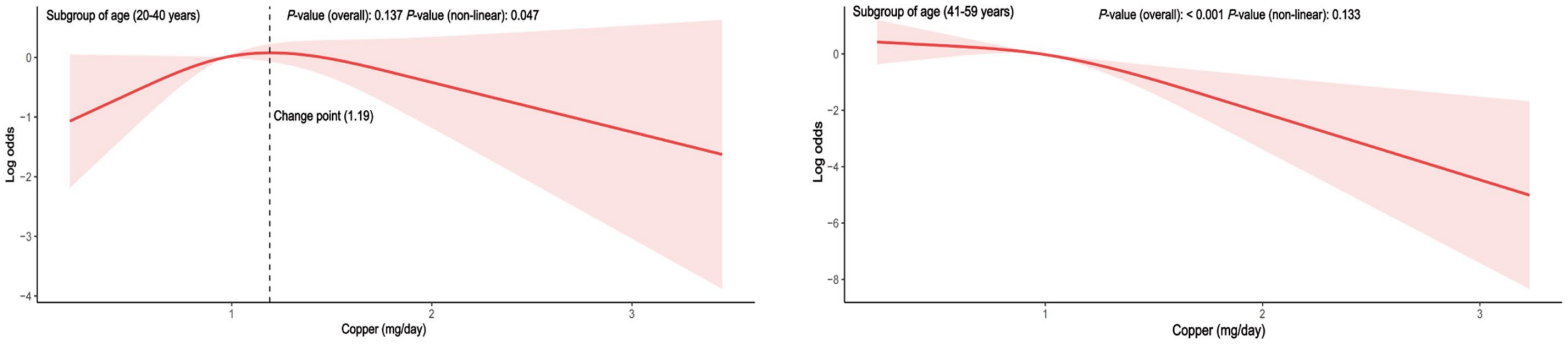
The RCS between dietary copper intake and the risk for PID in age-stratified subgroups (adjusted for all covariates except age).

**Figure 5:**
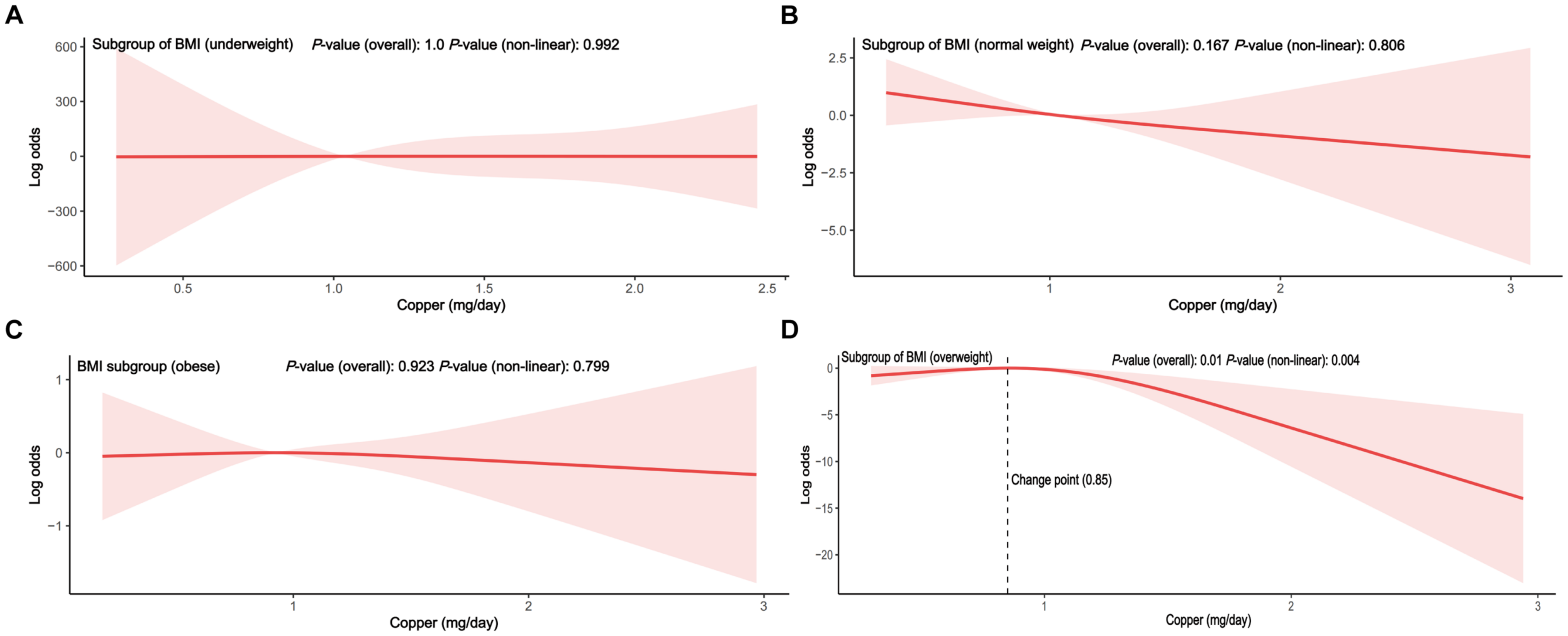
The RCS between dietary copper intake and the risk for PID in BMI stratified subgroup (adjusted for all covariates except BMI). **(A)** subgroup of underweight; **(B)** subgroup of normal weight; **(C)** subgroup of obesity; **(D)** subgroup of overweight.

To reduce heterogeneity, subgroup analyses were conducted, and the interaction between covariates and dietary copper intake was further examined (Figure 3). Here, nearly all ORs in the subgroup were lower than one except for the women participants in the underweight group, indicating a consistent negative relationship between copper intake and PID. Copper intake was positively related to PID in the underweight subgroup (OR = 1.61, 95% CI = 0.81–3.19), although without a statistically significant difference (*p* = 0.15). Interaction effects were also shown to be statistically significant for age (*p* for interaction = 0.01) and BMI (*p* for interaction = 0.01). To investigate the influence of meaningful interactions, RCS was conducted for better visualization in model 2 (all covariates were adjusted except for the grouping variable) (Figures 4, 5). Age-stratified analyses revealed a parabolic association between PID and copper intake (overall *p* value = 0.137, non-linear *p* value <0.05). In the age subgroup of 20–40 years old, the correlation between dietary copper intake and PID increased before the 1.19 mg/day point and then decreased gradually. In contrast, in the 41–59-year-old subgroup, a linear pattern of correlation was observed between dietary copper intake and PID (overall *p*-value <0.01, non-linear *p*-value = 0.13). After stratifying by BMI, copper intake was shown to significantly correlate with PID in the overweight subgroup (overall *p*-value = 0.01, non-linear *p*-value <0.01). The association between dietary copper intake and PID remained relatively stable until around 0.85 mg/day and then decreased rapidly. However, no significant *p*-values were observed in the underweight (overall *p*-value = 1.0, non-linear *p*-value = 0.99), normal weight (overall *p*-value = 0.17, non-linear *p*-value = 0.81), or obese subgroups (overall *p*-value*p* = 0.92, non-linear *p*-value = 0.80).

## 4 Discussion

PID is a chronic and recurrent disease that significantly impacts the well-being of patients, causing considerable suffering. Until now, few studies have been done on the dietary recommendations for women with PID. This is the first research to our knowledge to investigate the link between PID and dietary trace minerals. Based on the large sample size of the NHANES, dietary copper intake was shown to exhibit a significant negative linear relationship with PID; however, no difference was found in the correlation between other trace minerals (iron, selenium, and zinc) and PID. In addition, we found that age and BMI may have meaningful interactions between copper intake and PID. In the younger age subgroup, the relationship exhibited a curved pattern; however, in the older age subgroup, it showed a straight line. Similarly, a curved relationship was observed in the overweight subgroup.

Currently, dietary iron intake is mainly focused on pregnancy and anemia among women (25, 26). However, it has been shown that dietary iron intake exhibited no relationship with non-pregnancy diseases such as gynecological cancers (15). In our study, dietary iron intake was not significantly different between the PID and non-PID groups, as supported by insignificant results from logistic regression analysis. Selenium is now recognized as an important trace nutrient with anti-inflammatory function (27). Compared with the control group, in men, selenium supplementation improved inflammatory parameters (28). No significant link was found between selenium in the diet and PID in this study, which may be explained by sex differences and disease-specific factors. Zinc deficiency is closely related to an increased risk to pregnancy health because of its vital role in some enzymatic reactions (29), and bone mineral density is associated with zinc intake in a positive manner (30). However, in the present study, univariate analysis of dietary zinc intake and PID showed no significant relationship, suggesting avenues for future research. Compared to non-pregnant women, the urine levels of trace element silicon in pregnant women were higher, which might be accounted for by the differences in the gastrointestinal tract, kidney excretion, and metabolism in pregnant women (31). The trace elements metabolism also changed between gestational weeks (32). Consequently, as a non-pregnancy disease, PID might not be easily susceptible to changes in common trace elements.

Copper performs an integral function in the secretion of inflammatory products and is linked to many inflammatory diseases by regulating the nuclear factor kappa-B and mitogen-activated protein kinase pathways (14). Another study has shown that copper is capable of activating nicotinamide adenine dinucleotide phosphate hydrogen in mitochondria, thus mediating metabolic and epigenetic processes toward the inflammatory state (33). The dietary copper intake was demonstrated to be correlated with inflammatory disorders and serum estradiol levels in women (34, 35). Adequate copper intake is vital to maintain the antioxidant capacity of the body (36). Although, to our knowledge, no study has been conducted on dietary copper intake and PID, evidence has shown that using non-hormonal copper-containing intrauterine devices could effectively decrease pelvic pain, which highlights the importance of the copper mechanism in PID (37). Our data showed a consistent negative linear correlation between copper intake and PID, which remained stable across different models. Our findings imply that copper intake can aid in the fight against PID, which may contribute to its regulatory role in inflammation. However, a key consideration is that excessive copper intake may be harmful to health. Therefore, future research is needed to determine the specific turning points of copper intake that are effective in preventing and managing PID.

Age and body weight were independently associated with pelvic disease, especially in older and obese women (38). In our study, age and BMI covariates were identified as interaction terms between copper intake and PID. In the younger age group, it appears that adequate copper intake (>1.19 mg/day), which exceeds the RDA, may have a protective effect against PID. However, in the older age group, the relationship between copper intake and PID is assumed to be linear from the beginning of copper intake. According to a disease burden analysis, women younger than 25 years old had a higher rate of PID than older women (39). Young women may be more susceptible to the modulatory effects of high estrogen and progesterone levels, which in turn makes them less susceptible to the influence of copper intake. Among the different BMI subgroups, only the overweight participants showed a significant non-linear relationship between copper intake and PID, while no significant correlations were found in other BMI subgroups. Although there was an initially positive correlation between copper intake and PID in the underweight subgroup, this relationship disappeared in the subsequent multivariate RCS, which could be due to the presence of confounding variables. Before reaching a copper intake of 0.85 mg/day (close to the RDA), the link between copper intake and PID in the overweight subgroup (≥30.0 kg/m^2^) was flat and decreased rapidly when the RDA of copper was reached. A retrospective study has shown that obese PID patients were identified to be associated with an unfavorable clinical course (9). Thus, adequate copper intake may become even more important for older and overweight women with PID. Poultry was considered a valuable food for its abundance of minerals (specifically iron, zinc, and copper) and moderate energy (40). Therefore, intake of a certain amount of meat rich in copper may be beneficial for women with PID. However, the trace minerals consumed are not equivalent to the actual uptake of minerals, and the absorption efficiency may also be a critical factor. Before being absorbed, dietary copper (Cu^2+^) was first reduced to cuprous copper, then absorbed by the intestinal epithelial cells, and finally transported to the liver for processing and activation (41). Thus, people with gastrointestinal disease or liver diseases may present a low absorption efficiency of dietary copper. Furthermore, copper imbalance was associated with liver pathological features, including oxidative stress and mitochondrial dysfunction, which in turn affect the copper absorption efficiency (42). Hence, future studies on dietary copper absorption efficiency and PID will be required to apply our findings to real-world situations.

To our knowledge, our research is the first to investigate whether PID is linked to dietary trace minerals. A significant negative relationship between PID and dietary copper intake was identified, though not in iron, selenium, and zinc. Additionally, multiple linear regression was used to verify the solid relationship between dietary copper intake and PID after adjusting for several covariates. Our study may have important implications regarding the setting of recommended dietary copper intake for women with PID. However, there were several limitations to this research. To begin with, this was a cross-sectional study; therefore, no causal relationship could be determined based on the accurate dietary trace intake recommendation for women with PID. Second, the diagnostic nature of the PID questionnaire inevitably introduced some degree of selection bias. Thirdly, although efforts were made to incorporate related confounding factors, we were unable to address all confounders. Finally, sampling errors inherent in the NHANES data cannot be ruled out. Given these limitations, large prospective cohort studies are necessary to validate the link between dietary copper intake and determine the recommended intake of dietary trace minerals for women with PID.

## 5 Conclusion

Overall, our study suggested that decreased dietary copper intakes are linked to PID. However, no significant link was found for other dietary trace minerals (iron, selenium, and zinc). To confirm our findings, more large-scale prospective investigations are needed.

## Data availability statement

The original contributions presented in the study are included in the article/supplementary material, further inquiries can be directed to the corresponding authors.

## Ethics statement

The studies involving humans were approved by National Center for Health Statistics Ethics Review Board gave approval for the project (https://www.cdc.gov/nchs/nhanes/irba98.htm). The studies were conducted in accordance with the local legislation and institutional requirements. The participants provided their written informed consent to participate in this study.

## Author contributions

PH: Writing – original draft. SM: Conceptualization, Writing – original draft. HL: Data curation, Conceptualization, Writing – review & editing. XY: Data curation, Formal analysis, Software, Writing – review & editing. XZ: Conceptualization, Data curation, Supervision, Writing – review & editing. QZ: Supervision, Funding acquisition, Writing – review & editing.
